# Pan-Cancer Analyses Reveal Prognostic Value of Osteomimicry Across 20 Solid Cancer Types

**DOI:** 10.3389/fmolb.2020.576269

**Published:** 2020-11-05

**Authors:** Changsheng Yang, Hehai Pan, Lujun Shen

**Affiliations:** ^1^Department of Spine Surgery, The Third Affiliated Hospital of Southern Medical University, Orthopaedic Hospital of Guangdong Province, Guangzhou, China; ^2^Guangdong Provincial Key Laboratory of Bone and Joint Degeneration Diseases, Southern Medical University, Guangzhou, China; ^3^Guangdong Provincial Key Laboratory of Malignant Tumor Epigenetics and Gene Regulation, Sun Yat-sen Memorial Hospital, Sun Yat-sen University, Guangzhou, China; ^4^Breast Tumor Center, Sun Yat-sen Memorial Hospital, Sun Yat-sen University, Guangzhou, China; ^5^Department of Minimally Invasive Interventional Therapy, Sun Yat-sen University Cancer Center, Guangzhou, China; ^6^Collaborative Innovation Center for Cancer Medicine, State Key Laboratory of Oncology in South China, Sun Yat-sen University, Guangzhou, China

**Keywords:** osteomimicry, SPARC, SPP1, BGLAP, pan-cancer analyses, prognosis

## Abstract

**Background:**

Osteomimicry of cancer cells had been widely reported in prostate cancer and breast cancer. However, the prognostic value of osteomimicry in various cancer types remained unclear. We hypothesized that osteomimicry would result in remodeling of the tumor microenvironment and was eligible to predict patient prognosis.

**Methods:**

A comprehensive transcriptomic analysis of the osteomimicry, which was characterized by mRNA expression of SPARC, SPP1, and BGLAP, across 20 solid tumors (7564 patients) using RNA-seq data from The Cancer Genome Atlas (TCGA) was conducted. Samples of each cancer type were classified into subgroups (high vs. low) based on median value of osteomimetic markers, the associations of these markers with clinical outcomes, immune cell infiltration and immune checkpoints expression were explored.

**Results:**

Each osteomimetic marker harbored prognostic value in the pan-cancer analyses [SPARC: hazard ratio (HR) = 1.10, *p* = 0.028; SPP1: HR = 1.25, *p* < 0.001; BGLAP: HR = 1.13, *p* = 0.005]. Patients with high expression of all the three genes also had significantly unfavorable survival (HR = 1.61, *p* < 0.0001) compared with those of low expression. Correlation analyses demonstrated that osteomimicry was closely related to tumor purity, dendritic cells (DC) infiltration and expression of immune checkpoints.

**Conclusion:**

Osteomimicry had prognostic value in various cancer types and the underlying mechanism might correlate to the trapping and dysfunction of DCs in the tumor microenvironment, revealing the potential of osteomimicry as a target of immunotherapy.

## Introduction

Osteomimicry referred to the acquisition of genotypic and phenotypic properties of bone cells, predominantly osteoblast, in cancer cells (Rucci and Teti, 2018). It was reported that osteomimicry influenced all aspects of cancer development and metastasis including cell proliferation, adhesion, migration, invasion, and could promote cancer cell survival and angiogenesis (Kruger et al., 2014). Specially, it was considered that osteomimicry was closely related to cancer metastasis to skeleton.(Rucci and Teti, 2018; Shupp et al., 2018) Once reaching the bone, cancer cells with osteomimicry feature could produce factors that directly [i.e., RANKL (receptor activator of nuclear factor kappa B ligand), IL-2β, IL-6, IL-11, and TNF-α] or indirectly (PTHrP, parathyroid hormone-related peptide) promote osteoclastogenesis and bone resorption (Rucci and Teti, 2018).

However, the phenomenon of osteomimicry was mainly discussed in prostate cancer (Koeneman et al., 1999) and breast cancer (Croset et al., 2018; Wang et al., 2019). It was scarcely reported in other cancer types and whether it had prognostic value remained unknown. Thus, we conducted a pan-cancer transcriptomic analysis of osteomimicry across a broad spectrum of solid tumors to define its impact on cancer prognosis using large-scale RNA-sequencing (RNA-seq) data of The Cancer Genome Atlas (TCGA) tumor samples. Since osteonectin (ON, encoded by SPARC), osteopontin (OPN, encoded by SPP1) and osteocalcin (OCN, encoded by BGLAP) were typical bone matrix proteins expressed in the advanced stage of osteoblast differentiation,(Rucci and Teti, 2010, 2018) which indicated extracellular matrix mineralization (Shupp et al., 2018), they were selected as the markers of osteomimicry as reported in other literatures (Koeneman et al., 1999; Rucci and Teti, 2010). Besides, previous studies showed that SPP1 also mediated inflammatory responses by functioning as a chemoattractant for immune cells (Lamort et al., 2019), and SPARC was able to suppress the migration of dendritic cells (DCs) after antigen stimulation (Sangaletti et al., 2005). Thus, the relationship between osteomimicry and immune cell infiltration in the tumor microenvironment was also investigated in this study.

## Materials and Methods

### Dataset and Tumor Types

The dataset used consisted of RNA-seq data from TCGA tumor samples (data accessed in Jan 2020). All samples were assayed by RNA-seq, as described by the TCGA research Network. Gene expression values were represented as RNA-Seq by Expectation Maximization (RSEM) data normalized within each sample to the upper quartile of total reads.

Acute myeloid leukemia (LAML, *n* = 173) was excluded because it was hematologic tumor. Lower grade glioma (LGG, *n* = 530) and pheochromocytoma and paraganglioma (PCPG, *n* = 187) were excluded because they were not classically malignant tumors. Sarcoma (SARC, *n* = 265) was excluded because it was bone-derived tumor. Adrenocortical cancer (ACC, *n* = 79), bile duct cancer (CHOL, *n* = 45), large B-cell lymphoma (DLBC, *n* = 48), mesothelioma (MESO, *n* = 87), ocular melanomas (UVM, *n* = 80), uterine carcinosarcoma (UCS, *n* = 57) were excluded for the small sample size (*n* < 90).

Samples of 20 solid cancer types (*n* = 7564) were investigated in the final analyses, including bladder urothelial carcinoma (BLCA, *n* = 407), breast cancer (BRCA, *n* = 1095), cervical cancer (CESC, *n* = 302), colon and rectal adenocarcinoma (COAD, *n* = 283), esophageal cancer (ESCA, *n* = 184), glioblastoma (GBM, *n* = 160), head and neck squamous cell carcinoma (HNSC, *n* = 520), kidney clear cell carcinoma (KIRC, *n* = 533), kidney papillary cell carcinoma (KIRP, *n* = 290), liver hepatocellular carcinoma (LIHC, *n* = 371), lung adenocarcinoma (LUAD, *n* = 515), lung squamous cell carcinoma (LUSC, *n* = 501), pancreatic adenocarcinoma (PAAD, *n* = 178), prostate adenocarcinoma (PRAD, *n* = 497), rectal cancer (READ, *n* = 93), skin cutaneous melanoma (SKCM, *n* = 469), stomach adenocarcinoma (STAD, *n* = 380), thyroid cancer (THCA, *n* = 489), thymoma (THYM, *n* = 120), endometrioid cancer (UCEC, *n* = 177).

### Measurement of Osteomimicry

Osteomimicry was indicated by the gene expression of SPARC, SPP1, and BGLAP, using log 2-transformed values in RSEM. To further explore the effect of osteomimicry on patient’s prognosis and tumor microenvironment, patients were divided into high versus low subgroups according to median value of the expression of the osteomimetic markers in the corresponding cancer type.

### Evaluation of the Tumor Microenvironment

ESTIMATE (Estimation of STromal and Immune cells in MAlignant Tumor tissues using Expression data) (Yoshihara et al., 2013) was calculated to predict tumor purity.

The correlation between osteomimicry and immune cell infiltration in the tumor microenvironment was investigated by two independent tools, TIMER (tumor immune estimation resource)^1^ (Li et al., 2017) and ImmuCellAI^2^ (Miao et al., 2020).

### Gene Set Enrichment Analysis

To understand the differences in biological functions and pathways between different subgroups of osteomimicry, gene set enrichment analysis (GSEA^3^, accessed at January, 2020) was performed. We employed the molecular signatures Database (MSigDB) H (hallmark gene sets) collection of chemical and genetic perturbations (*n* = 20250 gene sets). Calculations were repeated 1000 times for each analysis according to the default weighted enrichment statistical method. GSEA results were shown using normalized enrichment scores (NES), accounting for the size and degree to which a gene set is overrepresented at the top or bottom of the ranked list of genes (nominal *p*-value < 0.05 and FDR ≤ 0.25).

### Statistical Analysis

Associations between subgroups and categorical variables (e.g., sex and disease stage) were analyzed using the chi-square test (Fisher’s exact test or Pearson’s chi-square test where appropriate), and the Mann–Whitney *U* test for continuous variables (e.g., age). Correlations between gene expression were evaluated using the spearman correlation test. The correlation was considered weak if the spearman coefficient was less than 0.2, moderate if less than 0.4, relatively strong if less than 0.6, strong if less than 0.8 and very strong if not less than 0.8. The prognostic significance was estimated using Kaplan-Meier survival curves. Cox proportional hazards model was used to calculate the hazard ration (HR) and corresponding 95% confidence interval (CI), incorporating age, sex and disease stage for adjustment. All statistical analyses were performed with R version 3.6.1. Statistical significance was set at *p* < 0.05 (two-sided).

## Results

### Characteristics of the Osteomimicry Across 20 Cancer Types

**FIGURE 1:**
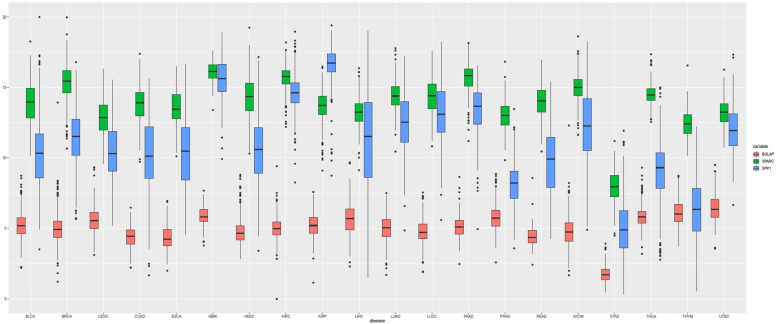
Boxplot of the gene expression (log_2_-transformed values) of SPARC, SPP1, and BGLAP according to TCGA cancer types.

A total of 7564 tumor samples from 20 TCGA cancer types were included. Figure 1 showed the log_2_-transformed values of SPARC/SPP1/BGLAP of different cancer types. The expression of SPARC and SPP1 were positively correlated in all the cancer types (*R*${}_{\text{overall}}^{2}$ = 0.54, *p* < 0.001) except KIRC (*R*^2^ = −0.14, *p* = 0.003), KIRP (*R*^2^ = −0.07, *p* = 0.237), THCA (*R*^2^ = 0.01, *p* = 1.000) and UCEC (*R*^2^ = 0.05, *p* = 0.470), but the correlation between the expression of BGLAP and SPARC/SPP1 was not prominent (*R*^2^ < 0.40 for all cancer types) (Table 1).

**TABLE 1:** Correlations between the expression level of BGLAP, SPARC, and SPP1 in different cancer types.

**BLCA**				**BRCA**				**CESC**				**COAD**				**ESCA**			
	BGLAP	SPARC	SPP1		BGLAP	SPARC	SPP1		BGLAP	SPARC	SPP1		BGLAP	SPARC	SPP1		BGLAP	SPARC	SPP1
BGLAP	1.00**	−0.02	0.00	BGLAP	1.00**	−0.01	−0.01	BGLAP	1.00**	−0.17**	0.02	BGLAP	1.00**	0.05	−0.01	BGLAP	1.00**	0.02	0.13
SPARC		1.00**	0.38**	SPARC		1.00**	0.22**	SPARC		1.00**	0.19**	SPARC		1.00**	0.69**	SPARC		1.00**	0.46**
SPP1			1.00**	SPP1			1.00**	SPP1			1.00**	SPP1			1.00**	SPP1			1.00**

**GBM**				**HNSC**				**KIRC**				**KIRP**				**LIHC**			

	BGLAP	SPARC	SPP1		BGLAP	SPARC	SPP1		BGLAP	SPARC	SPP1		BGLAP	SPARC	SPP1		BGLAP	SPARC	SPP1
BGLAP	1.00**	0.00	−0.08	BGLAP	1.00**	−0.09	−0.03	BGLAP	1.00**	−0.13**	−0.13**	BGLAP	1.00**	−0.24**	−0.18**	BGLAP	1.00**	−0.31**	−0.03
SPARC		1.00**	0.22*	SPARC		1.00**	0.30**	SPARC		1.00**	−0.14**	SPARC		1.00**	−0.07	SPARC		1.00**	0.14*
SPP1			1.00**	SPP1			1.00**	SPP1			1.00**	SPP1			1.00**	SPP1			1.00**

**LUAD**				**LUSC**				**PAAD**				**PRAD**				**READ**			

	BGLAP	SPARC	SPP1		BGLAP	SPARC	SPP1		BGLAP	SPARC	SPP1		BGLAP	SPARC	SPP1		BGLAP	SPARC	SPP1
BGLAP	1.00**	−0.10	−0.09	BGLAP	1.00**	−0.14**	−0.07	BGLAP	1.00**	−0.1	−0.11	BGLAP	1.00**	−0.13**	−0.21**	BGLAP	1.00**	−0.06	0.06
SPARC		1.00**	0.31**	SPARC		1.00**	0.21**	SPARC		1.00**	0.42**	SPARC		1.00**	0.29**	SPARC		1.00**	0.70**
SPP1			1.00**	SPP1			1.00**	SPP1			1.00**	SPP1			1.00**	SPP1			1.00**

**SKCM**				**STAD**				**THCA**				**THYM**				**UCEC**			

	BGLAP	SPARC	SPP1		BGLAP	SPARC	SPP1		BGLAP	SPARC	SPP1		BGLAP	SPARC	SPP1		BGLAP	SPARC	SPP1
BGLAP	1.00**	−0.07	−0.08	BGLAP	1.00**	0.03	0.01	BGLAP	1.00**	−0.01	−0.11*	BGLAP	1.00**	−0.27**	−0.21*	BGLAP	1.00**	−0.12	−0.31**
SPARC		1.00**	0.18**	SPARC		1.00**	0.41**	SPARC		1.00**	0.01	SPARC		1.00**	0.41**	SPARC		1.00**	0.05
SPP1			1.00**	SPP1			1.00**	SPP1			1.00**	SPP1			1.00**	SPP1			1.00**

*indicated *p* < 0.05. **indicated *p* < 0.01.

Overall, the median log_2_-transformed expression value of SPARC, SPP1 and BGLAP were 14.33 (interquartile range, [IQR], 13.17 to 15.38), 11.33 (IQR, 9.02 to 13.59) and 5.08 (IQR, 4.37 to 5.76), respectively. We then divided the samples into two subgroups (high vs. low) by the median value in each cancer type. The demographic and clinical features of the TCGA patients were summarized in Table 2. Generally, both age and gender distribution were similar between subgroups. Patients with tumor stage I/II were allocated into the early stage group and those with tumor stage III/IV were allocated into the advanced stage group. Osteomimicry was significantly associated with advanced tumor stage in BLCA, BRCA, CESC, COAD, HNSC, KIRC, and STAD, and was marginally significantly associated with advanced tumor stage in ESCA, LIHC, SKCM, and UCEC.

**TABLE 2:** Correlations between expression levels of osteomimetic markers and clinical features in different cancer types.

		**SPARC**						**SPP1**						**BGLAP**					
		**Age/year**	***p*-value**	**M/F**	***p*-value**	**Stage**	***p*-value**	**Age/year**	***p*-value**	**M/F**	***p*-value**	**Stage**	***p*-value**	**Age/year**	***p*-value**	**M/F**	***p*-value**	**Stage**	***p*-value**
BLCA	Up	69.30	0.028	146/57	0.367	37/166	<0.001	69.07	0.087	146/57	0.367	55/148	0.019	68.41	0.349	151/52	0.910	68/135	0.750
	Down	66.85		155/49		95/107		67.07		155/49		77/125		67.73		150/54		64/138	
BRCA	Up	57.74	0.074	6/541	na	388/144	0.158	59.18	0.129	6/541	na	409/122	0.121	58.28	0.463	5/542	na	416/120	0.041
	Down	59.15		6/542		414/125		57.72		6/542		393/147		58.61		7/541		386/149	
CESC	Up	47.49	0.565	0/151	na	113/34	0.675	49.79	0.033	0/151	na	106/40	0.034	47.56	0.277	0/151	na	112/35	0.485
	Down	48.83		0/151		117/31		46.53		0/151		124/25		48.76		0/151		118/30	
COAD	Up	65.35	0.704	80/61	0.633	78/63	0.400	65.46	0.586	80/61	0.633	73/68	0.040	64.18	0.295	75/66	0.551	71/70	0.011
	Down	64.77		76/66		86/56		64.66		76/66		91/51		65.93		81/61		93/49	
ESCA	Up	62.37	0.821	82/10	0.290	45/40	0.070	61.07	0.132	82/10	0.289	47/36	0.520	61.37	0.184	76/16	0.289	49/35	0.750
	Down	62.53		76/16		51/25		63.84		76/16		49/29		63.53		82/10		47/30	
GBM	Up	61.74	0.103	53/27	0.868	na	na	59.95	0.947	52/28	1.000	na	na	58.49	0.317	50/30	0.619	na	na
	Down	57.33		51/29				59.11		52/28				60.58		54/26			
HNSC	Up	60.51	0.611	189/71	0.617	55/175	0.360	61.89	0.056	192/68	1.000	41/188	0.039	60.94	0.995	202/58	0.057	46/170	0.732
	Down	61.23		195/65		43/172		59.85		192/68		57/159		60.80		182/78		52/177	
KIRC	Up	59.78	0.137	189/77	0.002	163/101	0.789	61.09	0.319	176/90	0.525	166/99	0.532	60.45	0.910	170/96	0.717	134/131	<0.001
	Down	61.47		156/111		161/105		60.17		169/98		158/107		60.80		175/92		190/75	
KIRP	Up	60.86	0.397	101/44	0.141	92/39	0.157	61.20	0.752	105/40	0.688	97/34	1.000	61.87	0.414	109/36	0.688	93/37	0.394
	Down	61.99		113/32		101/28		61.64		109/36		96/33		60.96		105/40		100/30	
LIHC	Up	58.05	0.033	119/66	0.224	126/44	1.000	60.18	0.259	127/58	0.658	119/52	0.066	58.63	0.161	125/60	1.000	129/47	0.806
	Down	60.83		131/55		131/46		58.70		123/63		138/38		60.25		125/61		128/43	
LUAD	Up	65.57	0.950	112/145	0.251	197/55	1.000	65.39	0.983	120/137	0.859	192/61	0.197	64.30	0.039	115/142	0.536	201/52	0.590
	Down	65.17		126/132		200/55		65.34		118/140		205/49		66.47		123/135		196/58	
LUSC	Up	68.03	0.015	186/64	0.918	205/42	0.487	67.73	0.210	195/55	0.052	202/47	0.816	67.45	0.452	183/67	0.684	196/51	0.202
	Down	66.37		185/66		201/49		66.68		176/75		204/44		66.96		188/63		210/40	
PAAD	Up	63.71	0.283	46/43	0.451	84/4	1.000	63.80	0.241	47/42	0.651	85/3	0.720	65.13	0.645	53/36	0.291	83/3	1.000
	Down	65.46		52/37		84/3		65.37		51/38		83/4		64.03		45/44		85/4	
PRAD	Up	61.72	0.026	248/0	na	na	na	61.34	0.212	248/0	na	na	na	61.20	0.707	248/0	na	na	na
	Down	60.33		249/0				60.70		249/0				60.84		249/0			
READ	Up	63.11	0.948	25/21	1.000	15/25	0.194	62.91	0.899	26/20	0.835	16/27	0.188	62.63	0.859	25/21	1.000	15/27	0.124
	Down	62.79		26/21		23/21		62.98		25/22		22/19		63.26		26/21		23/19	
SKCM	Up	56.86	0.115	143/91	0.849	118/99	0.771	56.26	0.008	139/95	0.343	107/104	0.066	57.22	0.149	144/90	1.000	123/93	0.498
	Down	59.50		146/89		120/94		60.13		150/85		131/89		59.14		145/90		115/100	
STAD	Up	64.71	0.437	130/60	0.280	85/97	1.000	65.66	0.241	122/68	0.666	99/84	0.006	64.87	0.523	127/63	0.666	86/97	1.000
	Down	65.55		119/71		86/98		64.60		127/63		72/111		65.38		122/68		85/98	
THCA	Up	46.17	0.080	77/175	0.071	168/83	1.000	47.90	0.422	73/179	0.317	160/92	0.129	47.33	0.930	74/178	0.229	166/85	0.776
	Down	48.36		59/194		168/84		46.64		63/190		176/75		47.21		62/191		170/82	
THYM	Up	61.46	0.002	32/28	1.000	na	na	58.29	0.658	35/25	0.272	na	na	58.63	0.411	35/25	0.272	na	na
	Down	54.37		31/29				57.48		28/32				57.15		28/32			
UCEC	Up	64.08	0.076	0/88	na	57/31	0.258	65.43	0.975	0/88	na	56/32	0.145	65.38	0.654	0/88	na	55/33	0.070
	Down	67.03		0/89		65/24		65.72		0/89		66/23		65.77		0/89		67/22	

*na indicated “not applicable.”*

### High Expression of SPARC/SPP1/BGLAP Was Associated With Unfavorable Prognosis

**FIGURE 2:**
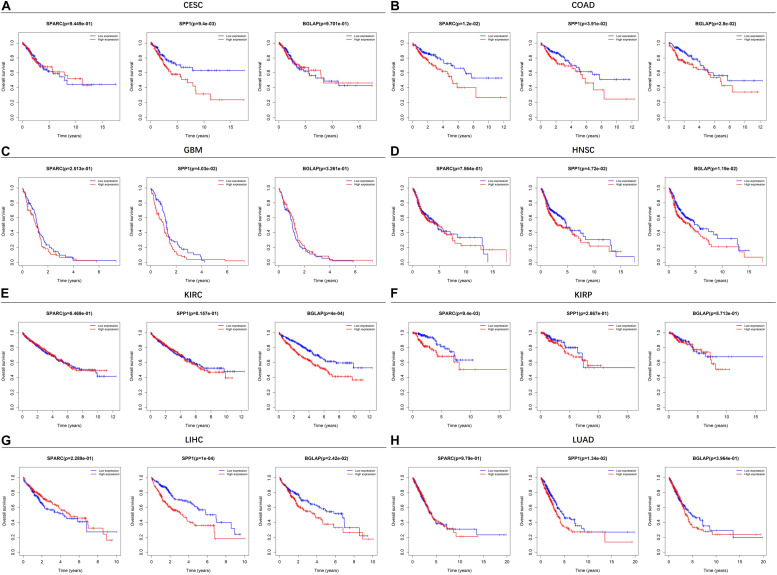
Kaplan-Meier plots of overall survival in different SPARC/SPP1/BGLAP subgroups in the cancer types in which they had prognostic value. **(A)** CESC, **(B)** COAD, **(C)** GBM, **(D)** HNSC, **(E)** KIRC, **(F)** KIRP, **(G)** LIHC, **(H)** LUAD. *p* < 0.05 represents significant difference in survival outcomes.

Figure 2 showed the results of survival analyses across the 8 cancer types in which SPARC/SPP1/BGLAP had prognostic value. The results of the other 12 cancer types were summarized in Supplementary Figure 1. Overall, patients in the high expression subgroups had relatively unfavorable clinical outcomes. While for each cancer type, the three markers harbored different prognostic values. Specifically, for SPARC, patients in the high expression subgroup had significantly unfavorable survival in COAD and KIRP, and had marginally significantly unfavorable survival in BLCA, LUSC, and STAD. For SPP1, patients in the high expression subgroup had significantly unfavorable survival in CESC, COAD, GBM, HNSC, LIHC, LUAD, and had marginally significantly unfavorable survival in LUSC and PAAD. For BGLAP, patients in the high expression subgroup had significantly unfavorable survival in COAD, HNSC, KIRC, and LIHC.

**FIGURE 3:**
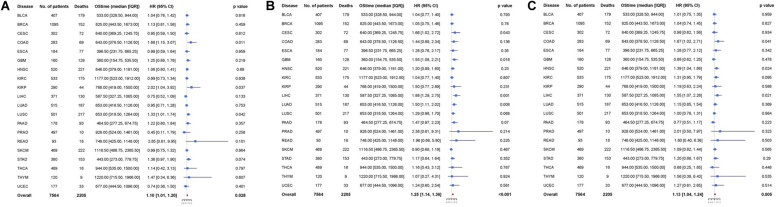
Cox proportional hazards analyses of **(A)** SPARC, **(B)** SPP1, and **(C)** BGLAP across 20 cancer types. Note: age (young vs. old, the median age as the threshold), sex (male vs. female) and disease stage (stage III-IV vs. stage I-II) were included in the multivariable model for adjustment. *p* < 0.05 represents significant difference in survival outcomes.

Multivariable analysis was performed to explore whether osteomimicry was a prognostic factor for survival outcomes, incorporating clinically relevant covariates (including tumor stage) for adjustment (Figure 3). The results of multivariable modeling largely supported the findings in the univariable analysis. Higher expression of SPARC was an unfavorable prognosticator for patients with COAD (HR = 1.88, 95%CI = [1.15, 3.07], *p* = 0.011), KIRP (HR = 2.02, 95% CI = [1.04, 3.92], *p* = 0.037), LUSC (HR = 1.33, 95% CI = [1.01, 1.74], *p* = 0.042), and had a trend of unfavorable survival in STAD (HR = 1.36, 95% CI = [0.97, 1.90], *p* = 0.074). Higher expression of SPP1 was an unfavorable prognosticator for patients with CESC (HR = 1.66, 95%CI = [1.02, 2.72], *p* = 0.043), GBM (HR = 1.55, 95% CI = [1.08, 2.21], *p* = 0.016), LIHC (HR = 1.88, 95% CI = [1.29, 2.75], *p* = 0.001), LUAD (HR = 1.50, 95% CI = [1.11, 2.02], *p* = 0.008), and had a trend of unfavorable survival in LUSC (HR = 1.29, 95% CI = [0.98, 1.70], *p* = 0.068), PAAD (HR = 1.47, 95% CI = [0.97, 2.23], *p* = 0.070). Higher expression of BGLAP was an unfavorable prognosticator for patients with COAD (HR = 1.67, 95%CI = [1.02, 2.71], *p* = 0.041), HNSC (HR = 1.39, 95% CI = [1.04, 1.86], *p* = 0.024), LIHC (HR = 1.55, 95% CI = [1.07, 2.26], *p* = 0.021), and had a trend of unfavorable survival in KIRC (HR = 1.31, 95% CI = [0.95, 1.79], *p* = 0.095).

Next, we pooled all the 20 cancer types and found that all the three markers were prognosticator of solid cancers (SPARC: HR = 1.10, 95% CI = [1.01, 1.20], *p* = 0.028; SPP1: HR = 1.25, 95% CI = [1.14, 1.36], *p* < 0.001; BGLAP: HR = 1.13, 95% CI = [1.04, 1.24], *p* = 0.005) (Figure 3).

### High Expression of All the Three Genes Indicated Unfavorable Prognosis

**FIGURE 4:**
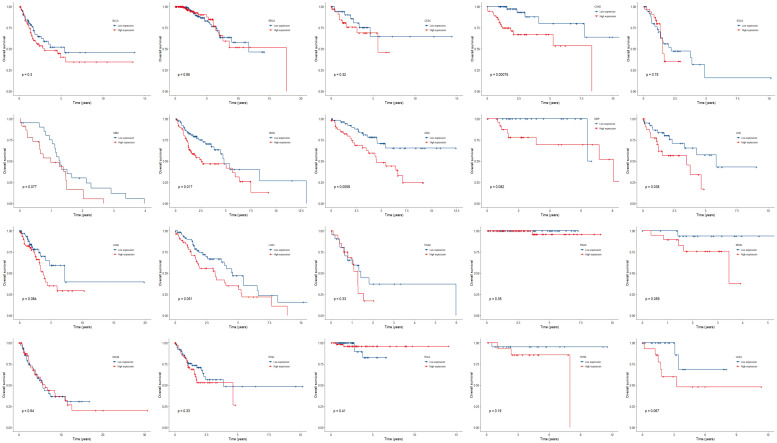
Kaplan-Meier plots of overall survival in subgroups divided by all the three genes across 20 cancer types. *p* < 0.05 represents significant difference in survival outcomes.

We further compared the survival outcome of patients with high expression of all the three genes with those with low expression (Figure 4). High expression subgroups had significant unfavorable survival in COAD, HNSC, KIRC, LIHC, and had marginally significantly unfavorable survival in GBM, KIRP, LUAD, LUSC, READ, and UCEC.

**FIGURE 5:**
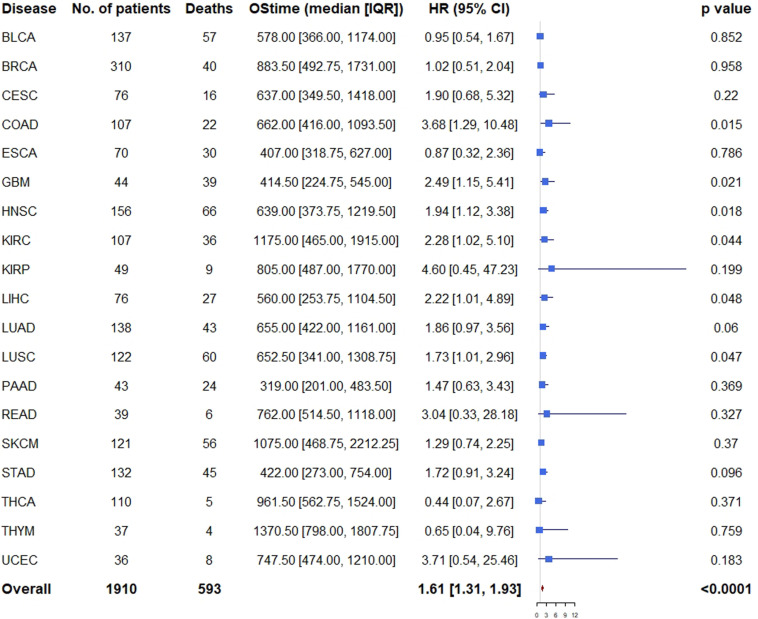
Cox proportional hazards analyses of all the three genes across 20 cancer types. Note: age (young vs. old, the median age as the threshold), sex (male vs. female) and disease stage (stage III-IV vs. stage I-II) were included in the multivariable model for adjustment. *p* < 0.05 represents significant difference in survival outcomes.

Similarly, multivariable analysis was also performed to explore whether patients with high expression of all the three genes had a different survival outcome. We found that patients with high expression of all the three genes had significantly unfavorable prognosis in COAD (HR = 3.68, 95% CI = [1.29, 10.48], *p* = 0.015), GBM (HR = 2.49, 95% CI = [1.15, 5.41], *p* = 0.021), HNSC (HR = 1.94, 95% CI = [1.12, 3.38], *p* = 0.018), KIRC (HR = 2.28, 95% CI = [1.02, 5.10], *p* = 0.044), LIHC (HR = 2.22, 95% CI = [1.01, 4.89], *p* = 0.048), LUSC (HR = 1.73, 95% CI = [1.01, 2.96], *p* = 0.047), and marginally significantly unfavorable prognosis in LUAD (HR = 1.86, 95% CI = [0.97, 3.56], *p* = 0.060) and STAD (HR = 1.72 95% CI = [0.91, 3.24], *p* = 0.096) (Figure 5).

As expected, when all the 19 cancer types (PRAD was excluded because there was only one death) were pooled, we found that patients with high expression of all the three genes had significantly unfavorable prognosis (HR = 1.61, 95% CI = [1.31, 1.93], *p* < 0.001) compared with those of low expression (Figure 5).

### Correlation Between Osteomimicry and Immune Cell Infiltration

It was reported that the osteomimicry may contribute to an immunosuppressive microenvironment in cancer (Reinstein et al., 2017). Therefore, the patterns of immune cell infiltration were evaluated in the cancer types in which osteomimicry indicated unfavorable survival.

**FIGURE 6:**
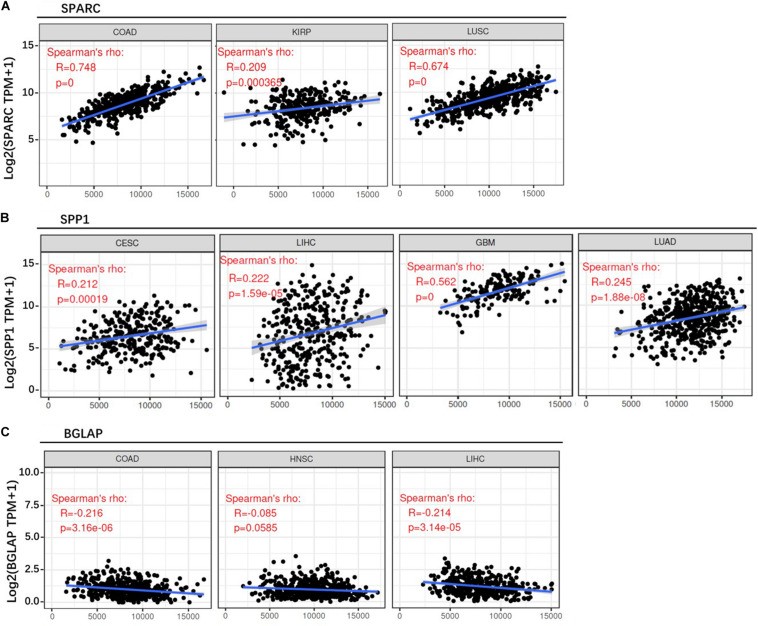
Correlation between osteomimicry and ESTIMATE score in the cancer types in which osteomimicry indicated unfavorable survival. SPARC **(A)** and SPP1 **(B)** expression were positively correlated to ESTIMATE score, but BGLAP **(C)** expression was weakly and negatively correlated to ESTIMATE score. *p* < 0.05 represents that difference is statistically significant.

First of all, we evaluated the correlation between osteomimetic markers and tumor purity. The result showed that SPARC and SPP1 expression were positively correlated to ESTIMATE score, but BGLAP expression was weakly and negatively correlated to ESTIMATE score (Figure 6).

**FIGURE 7:**
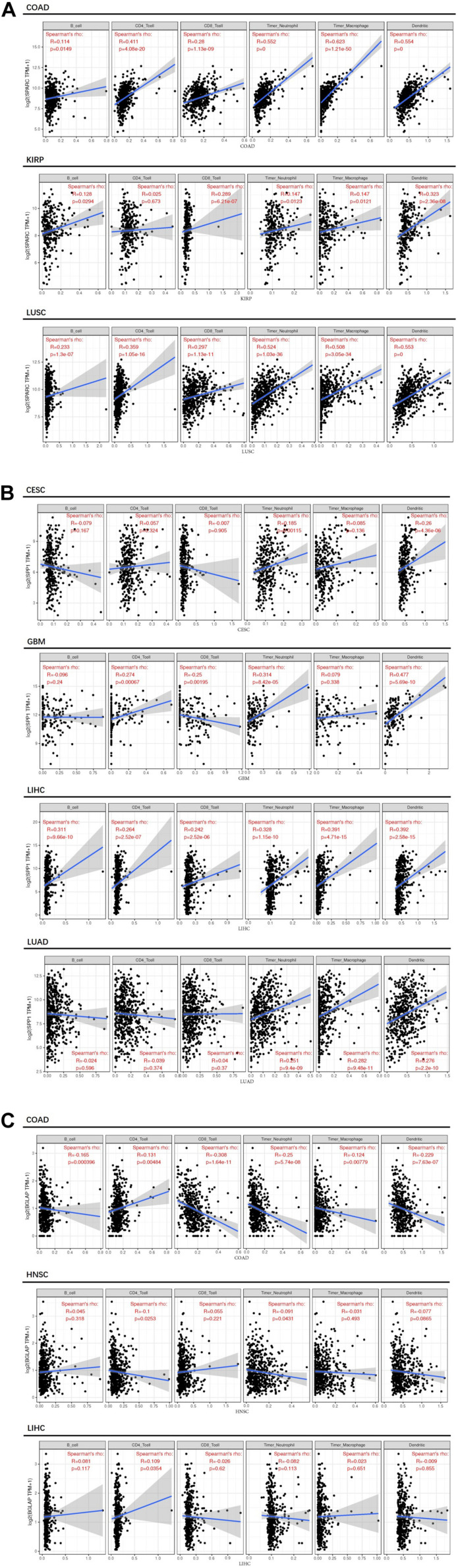
Correlation between osteomimicry and immune cell infiltration in the cancer types in which osteomimicry indicated unfavorable survival. Results from TIMER demonstrated that SPARC **(A)** and SPP1 **(B)** expression were positively associated with infiltration of DCs and neutrophil cells in all study models. BGLAP **(C)** expression showed no prominent correlation with immune cell infiltration. *p* < 0.05 represents that difference is statistically significant.

**FIGURE 8:**
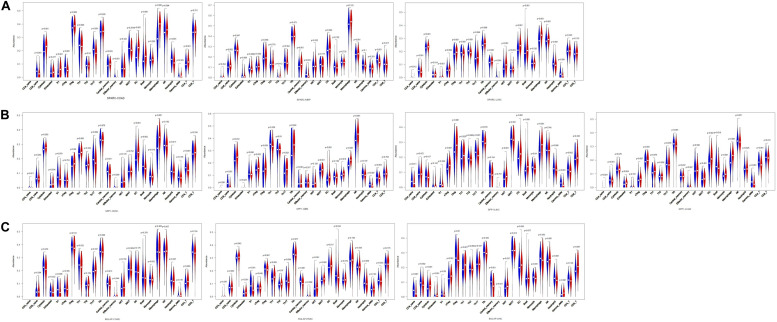
Violin plots of comparison of immune cell infiltration between high and low subgroups in the cancer types in which osteomimicry indicated unfavorable survival. Results from ImmuCellAI indicated that the high expression subgroups of SPARC **(A)**, SPP1 **(B)**, and BGLAP **(C)** had significant more DCs infiltration in the majority of study models. *p* < 0.05 represents that difference is statistically significant.

Second, we investigated the correlation of osteomimicry with tumor-infiltrating immune cells via two independent methods. Results from TIMER demonstrated that SPARC and SPP1 expression were positively associated with infiltration of DCs and neutrophil cells in all the study models (Figure 7). However, no prominent correlation was found between BGLAP expression and immune cell infiltration (*R*^2^ < 0.40 for all cancer types and all the six immune cell types) (Figure 7). While using ImmuCellAI, we compared 24 infiltrative immune cell types between subgroups, and found that the high expression subgroups had significant more DCs infiltration in the majority of study models (Figure 8).

### Correlation Analysis Between Osteomimicry and Immune Checkpoint Expression

**FIGURE 9:**
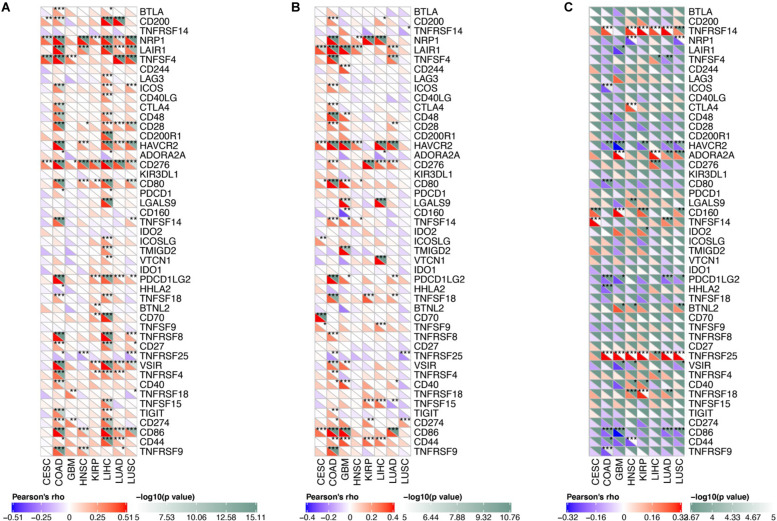
Correlation between osteomimetic markers **(A)** SPARC, **(B)** SPP1 and **(C)** BGLAP and immune checkpoints in the cancer types in which osteomimicry indicated unfavorable survival. SPARC **(A)** and SPP1 **(B)** expression were positively correlated to DC-related markers CD86, NRP1 and inhibitory checkpoint markers including LAIR1, HAVCR2 and PDCDLG2 in most cancer types. SPARC **(A)** expression was positively and significantly correlated to CD276 (B7-H3) in all the eight cancer types. BGLAP **(C)** expression was positively and significantly correlated to TNFRSF25 in all these cancer types except CESC. *indicated *p* < 0.05. **indicated *p* < 0.01. ***indicated *p* < 0.001. *p* < 0.05 represents that difference is statistically significant.

We analyzed the correlation between osteomimicry and immune checkpoint expression in the eight cancer types in which osteomimicry harbored prognostic value. All the three markers were found to be correlated to the expression of a series of immune checkpoints. Particularly, SPARC and SPP1 expression were positively correlated to DC-related markers CD86, NRP1, and inhibitory checkpoint markers including LAIR1, HAVCR2, and PDCDLG2 in most cancer types. SPARC expression was positively and significantly correlated to CD276 (B7-H3) in all the eight cancer types. BGLAP expression was positively and significantly correlated to TNFRSF25 in all these cancer types except CESC (Figure 9).

### Gene Set Enrichment Analyses of Osteomimetic Expression Subgroups

GSEA was performed to explore the potential biological pathways of osteomimicry. We chose COAD (SPARC), LUSC (SPARC), CESC (SPP1), GBM (SPP1), LIHC (SPP1), LUAD (SPP1), HNSC (BGLAP), and LIHC (BGLAP) as the study models, in which the osteomimicry influenced both prognosis and immunological features in these tumors. The common enriched gene sets in the high expression subgroups included unfolded protein response and glycolysis. Under the conditions of FDR < 25% and *p*-value < 5%, for SPARC, there were 35 and 27 enriched gene sets in the high expression subgroup of COAD and LUSC, respectively, for SPP1, the gene set P53 pathway was enriched in the high expression subgroup of CESC, GBM, LIHC, for BGLAP, the gene set of DNA repair was enriched in the high expression subgroup of HNSC and LIHC.

## Discussion

Here, we presented several key aspects of the tumor osteomimicry, identified from RNA-seq data across large-scale TCGA solid tumor samples. Although the concept of osteomimicry was scarcely reported in cancer types other than prostate cancer and breast cancer, we observed the expression of bone markers in all of the twenty cancer types included in our study, indicating osteomimicry might be a common phenomenon in tumor pathophysiology. To our knowledge, this was the first comprehensive transcriptomic investigation of the osteomimetic expression across a large spectrum of solid tumors.

We divided the samples into subgroups based on the median value of each osteomimetic marker and investigated their roles in predicting survival outcomes. Generally, subgroups with high expression of osteomimetic markers had more advanced tumor stage in most of the cancer types. Accordingly, survival analysis showed that the osteomimicry harbored prognostic value in many cancer types, including CESC (SPP1), COAD (SPARC, SPP1, BGLAP, SPARC + SPP1 + BGLAP), GBM (SPP1), HNSC (SPP1, BGLAP, SPARC + SPP1 + BGLAP), KIRC (BGLAP, SPARC + SPP1 + BGLAP), KIRP (SPARC), LIHC (SPP1, BGLAP, SPARC + SPP1 + BGLAP), LUAD (SPP1). However, we failed to analyze the relation between osteomimicry and bone metastasis for lack of information about metastasis site in the raw data.

Further analysis demonstrated that the osteomimicry remained a prognosticator even after including disease stage for adjustment in CESC (SPP1), COAD (SPARC, BGLAP, SPARC + SPP1 + BGLAP), GBM (SPP1, SPARC + SPP1 + BGLAP), HNSC (BGLAP, SPARC + SPP1 + BGLAP), KIRC (SPARC + SPP1 + BGLAP), KIRP (SPARC), LIHC (SPP1, BGLAP, SPARC + SPP1 + BGLAP), LUAD (SPP1) and LUSC (SPARC, SPARC + SPP1 + BGLAP), and harbored marginally significant prognostic value in BLCA (SPARC), LUAD (SPARC + SPP1 + BGLAP), PAAD (SPP1) and STAD (SPARC, SPARC + SPP1 + BGLAP).

There were some reports in regard to the relation between SPARC/SPP1/BGLAP and the prognosis of solid tumors. Ma et al. (2019) performed a systematic review recently and concluded that higher SPARC was associated with poor prognosis in gastrointestinal tumors (gastric cancer, esophageal squamous cell carcinoma, colorectal cancer, and biliary tract cancer), pancreatic cancer and respiratory tract tumors (non-small cell lung cancer and nasopharyngeal carcinoma). Higher SPP1 expression had been reported to be correlated to poor prognosis in liver cancer, urothelial carcinoma, breast cancer, colorectal cancer (Zhao et al., 2018). Lewenhaupt et al. found serum BGLAP level was associated with poor prognosis in prostate cancer (Ekman et al., 1988; Lewenhaupt et al., 1988, 1990). However, in previous studies, SPARC/SPP1/BGLAP was deemed independent functional molecule rather than marker of osteomimicry, and was investigated separately in different cancer types and no one had combined these molecules and explored their prognostic value in pan-cancer analyses. In our study, for the first time to our knowledge, we revealed that high expression of these osteomimetic markers was associated with unfavorable survival across a large spectrum of solid tumors. Furthermore, to better distinguish osteomimicry, patients were divided into subgroups with high versus low expression of all these three genes. Similarly, subgroups of high expression of all the three genes had worse survival, and showed higher HR (1.61) than that calculated with only one marker (1.10 for SPARC, 1.25 for SPP1, 1.13 for BGLAP). The above observations further revealed the importance of osteomimicry in the prediction of prognosis of various cancer types.

Tumor microenvironment has long been shown playing an important role in cancer progression, response to clinical intervention including immunotherapy, and clinical outcome of cancer patients (Schelker et al., 2017; Binnewies et al., 2018; Deng et al., 2020). However, whether osteomimicry had any impact on the tumor microenvironment remained obscure. The correlation between osteomimicry and tumor purity was then investigated, and result showed that SPARC and SPP1 expression had a moderate-to-strong and positive correlation with tumor purity, while BGLAP expression had a poor-to-moderate and negative correlation with tumor purity. This was consistent with the process of osteoblast differentiation, which could be divided into three stages: proliferation, extracellular matrix maturation, and extracellular matrix mineralization. SPARC, SPP1, and BGLAP genes were sequentially expressed in the three stages of osteoblast differentiation (Shupp et al., 2018). High SPARC expression was observed in newly mineralizing bone but not in osteoid, while increase in BGLAP synthesis was observed during the transition from osteoid to mature mineralized matrix (Nakase et al., 1994; Licini et al., 2019). Therefore, higher BGLAP expression might indicate complete remodeling of the tumor microenvironment and cause a relatively low tumor purity.

Further, we investigated the immune cell infiltration in the tumor microenvironment using two different databases. Interestingly, the results from both TIMER and ImmuCellAI demonstrated that patients with a high osteomimetic expression harbored a high intratumoral DCs infiltration. DCs had been known as the antigen presenting cells and were able to prime, activate and direct the T cells to target tumor cells (Saxena and Bhardwaj, 2018). Previous reports had demonstrated that SPARC and SPP1 were able to suppress the migration of DCs to lymph nodes, which would result in the accumulation of DCs at primary tumor site (Sangaletti et al., 2005; Begum et al., 2007). Recent studies also showed that local factors within tumor microenvironment, including hypoxia, adenosine and lactate accumulations and decreased pH, could lead to dysfunction of DCs which were trapped at primary tumor site (Veglia and Gabrilovich, 2017). IL-10 produced by tumor-associated-macrophage could also inhibit IL-12 production by DCs and alter their ability to mount antigen specific T cell responses (Ruffell et al., 2014). The higher expression of inhibitory molecules (such as PD-L1) (Salmon et al., 2016) and accumulating lipid (Herber et al., 2010) in the tumor microenvironment could also lead to the dysfunction of DCs. It was reported that DCs with abnormal function in the tumor microenvironment can produce tumor promoting IL-6 and immunosuppressive galectin-1, which can impair the local anti-tumor immunity (Tesone et al., 2016). Therefore, it was reasonable to speculate that osteomimicry might trap the DCs at primary tumor site, leading to dysfunction and even immunosuppression of DCs and thus a poor prognosis of patients.

Certainly, the bone matrix protein SPARC/SPP1/BGLAP were functional molecules and played an important role in various malignancies according to previous literatures. Besides regulating bone mineralization, SPP1 has also been shown playing a role in tumor cell proliferation, adhesion, invasion and metastasis (Zhao et al., 2018; Pang et al., 2019). It was reported that SPP1 could regulate MMP-2 and MMP-9 expression and promote extracellular matrix degradation via activating αvβ-NF-κB pathway, and thus accelerate the growth, migration and invasion of cancer cells (Fong et al., 2009; Liu et al., 2010; Qin et al., 2018). Some studies also showed that SPP1 contributed to tumor cell migration, invasion and survival through PI3K-Akt (Fong et al., 2009; Zhang et al., 2014; Wei et al., 2018) and STAT3 (Behera et al., 2010) signaling, and could also promote tumor growth and metastasis by inducing angiogenesis (Dai et al., 2009; Wang et al., 2011). SPARC was a matricellular protein modulating cell-matrix interactions and was found up-regulated in tumor stroma and associated with poor prognosis in many cancer types, including colorectal cancer (Kurtul et al., 2017; Drev et al., 2019), pancreatic cancer (Vaz et al., 2015), cervical carcinoma (Shi et al., 2016), and non-small cell lung cancer (Koukourakis et al., 2003). It’s worth noting that the influence of SPARC on tumor progression depended on the initiating cell type, tumor stage and context of the microenvironment (Tai and Tang, 2008; Arnold and Brekken, 2009; Rossi et al., 2016). Notably, Munasinghe et al. reported that depletion of fibronectin switched the function of SPARC from promoting cancer cell proliferation to growth inhibition and induction of apoptosis (Munasinghe et al., 2020). Although BGLAP was known crucial during bone remodeling, the function remained largely unknown and it arose great interest recently as a bone-derived hormone in regulation of energy metabolism (Li et al., 2016; Zoch et al., 2016; Mera et al., 2018; Moser and van der Eerden, 2018).

Among the solid tumors investigated in this study, osteomimicry was found unable to predict prognosis in some cancer types. Although osteomimicry was used to explain the predilection of skeleton metastasis in prostate cancer and breast cancer, it showed no correlation with advanced tumor stage or poor survival in PRAD in this study. BGLAP was correlated to advanced tumor stage in BRCA, but showed no prognostic value. The failure to detect association might due to lack of adequate follow-up time, limited event rates, biased population distribution or medical intervention.

The main limitation of this study is lack of ability of osteomimicry to predict the response to immunotherapy, and it requires further validation in cancer patients treated with immune checkpoint inhibitors. Nevertheless, our findings are important and provide new insights into the prognostic and immunological features of osteomimicry in various solid tumors. Future molecular studies are warranted to shed light on how osteomimicry affects different cancers and survival outcomes.

## Data Availability Statement

The original contributions presented in the study are included in the article/Supplementary Material, further inquiries can be directed to the corresponding author/s.

## Ethics Statement

Ethical review and approval was not required for the study on Human participants in accordance with the Local Legislation and Institutional Requirements. Written informed consent from the participants’ legal guardian/next of kin was not required to participate in this study in accordance with the National Legislation and the Institutional Requirements.

## Author Contributions

CY designed this study and contributed to data collection, data analysis, data interpretation, and writing the manuscript. LS helped to design this study and contributed to data collection, data analysis and data interpretation, and revising the manuscript. HP helped to design this study and contributed to data interpretation and revising the manuscript. All authors contributed to the article and approved the submitted version.

## Conflict of Interest

The handling Editor declared a shared affiliation, though no other collaboration, with one of the authors CY.

## Footnotes

1 https://cistrome.shinyapps.io/timer/

2 http://bioinfo.life.hust.edu.cn/web/ImmuCellAI/

3 http://software.broadinstitute.org/gsea/index.jsp

## Supplementary Material

Supplementary Figure 1 Kaplan-Meier plots of overall survival in different SPARC/SPP1/BGLAP subgroups in the cancer types in which they acquired no prognostic value. **(A)** BLCA, **(B)** BRCA, **(C)** ESCA, **(D)** LUSC, **(E)** PAAD, **(F)** PRAD, **(G)** READ, **(H)** SKCM, **(I)** STAD, **(J)** THCA, **(K)** THYM, **(L)** UCEC. *p* < 0.05 represents significant difference in survival outcomes.
