# Supplementary figures and images for: Pan-Cancer Analyses Reveal Prognostic Value of Osteomimicry Across 20 Solid Cancer Types
Source: Front Mol Biosci. 2020 Nov 5;7:576269. doi: 10.3389/fmolb.2020.576269 (PMC7678014; doi:10.3389/fmolb.2020.576269)

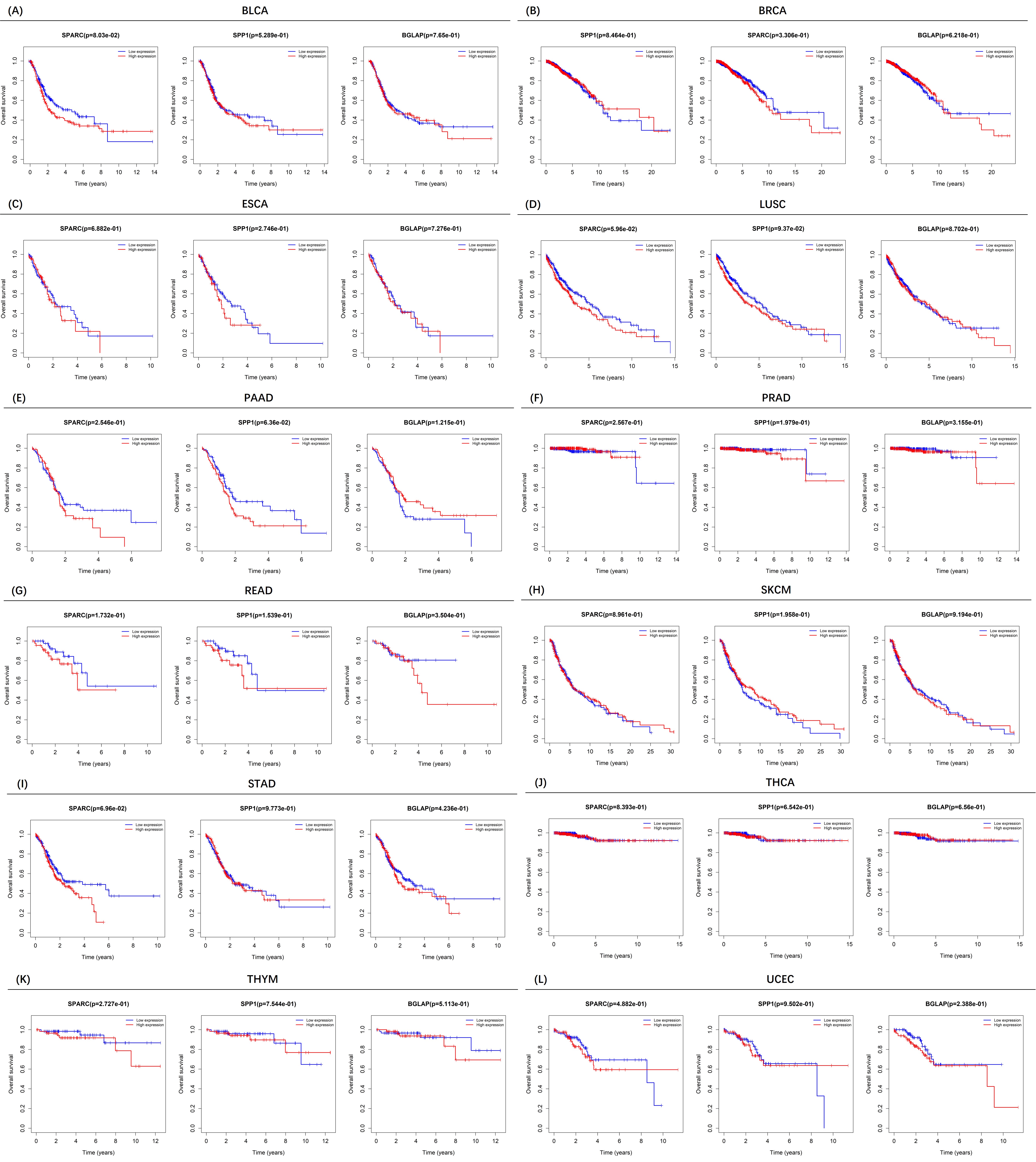

Supplement: Supplementary Figure 1 — Kaplan-Meier plots of overall survival in different SPARC/SPP1/BGLAP subgroups in the cancer types in which they acquired no prognostic value. (A) BLCA, (B) BRCA, (C) ESCA, (D) LUSC, (E) PAAD, (F) PRAD, (G) READ, (H) SKCM, (I) STAD, (J) THCA, (K) THYM, (L) UCEC. p < 0.05 represents significant difference in survival outcomes. [file Figure_1.JPEG]
